# The temperature dependence of amino acid hydrophobicity data is related to the genetic coding algorithm for complementary (sense and antisense) peptide interactions

**DOI:** 10.1016/j.dib.2020.105392

**Published:** 2020-03-07

**Authors:** Nikola Štambuk, Paško Konjevoda

**Affiliations:** a Centre for Nuclear Magnetic Resonance, Ruđer Bošković Institute, Bijenička cesta 54, HR-10000 Zagreb, Croatia; b Laboratory for Epigenomics, Division of Molecular Medicine, Ruđer Bošković Institute, Bijenička cesta 54, HR-10000 Zagreb, Croatia

**Keywords:** Genetic code, Amino acid, Hydrophobicity, Temperature, Peptide interaction

## Abstract

We present the data concerning the clustering of sense and antisense amino acid pairs into polar, nonpolar and neutral groups, as measured using hydrophobicity parameter—logarithmic equilibrium constants (Log_10_ K_w>c_)—at 25 °C and 100 °C (Wolfenden et al., 2015). The Log_10_ K_w>c_, values, of the complementary amino acid pairs are strongly correlated to the central (2nd) purine base of the mRNA codon and the complementary pyrimidine base of the tRNA anticodon. Clustering of amino acids is temperature independent with regard to the direction of translation (3′ → 5′ or 5′ → 3′). The Log_10_ K_w>c_ discriminate between artificial Hecht α- and β-protein datasets at 25 °C and 100 °C. Interpretation of this data may be found in the research article entitled “Determining amino acid scores of the genetic code table: complementarity, structure, function and evolution” (Štambuk and Konjevoda, 2020).

**Specifications table**

**Table:** 

Subject	Biochemistry, Genetics and Molecular Biology
Specific subject area	Structural biology: Analyses of protein structure and amino acid clustering with respect to codon complementarity and temperature parameter
Type of data	1, Spreadsheet data in CSV format, Supplementary Table S1 and Supplementary Table S2. Those data contain sequences of 15 artificial Hecht α- and 17 β-protein folds converted into a numerical series by assigning hydrophobicity parameter, logarithmic equilibrium constants (Log_10_ K_w>c_)—at 25 °C and 100 °C, to each amino acid 2, Table listing correlations of complementary amino acid pairs, in both translation directions, with respect to the logarithmic equilibrium constants (Log10 K_w>c_)—at 25 °C and 100 °C 3, Figures showing relationships between values given in Table and the text
How data were acquired	The datasets of 32 artificial Hecht proteins, 15 α- and 17 β-folds, in Supplementary Table S1 and Supplementary Table S2, consist of protein sequences converted into a numerical series, by assigning logarithmic equilibrium constants (Log_10_ K_w>c_) to each amino acid at 25 °C and 100 °C.
Data format	Raw and analyzed data
Parameters for data collection	The parameters for numerical conversion of sequences and statistical analyses were selected as described and given in the original article.
Description of data collection	Datasets of 15 artificial α- and 17 β-protein folds, expressed as a numerical series, were obtained by assigning the values of logarithmic equilibrium constants (Log_10_ K_w>c_ at 25 °C and 100 °C) to each amino acid of the sequence. This is a “gold standard” artificial protein dataset that could be successfully used both to test current methods and to develop new ones for the characterization of artificially-designed molecules based on the specific binary patterns of amino acid polarity (Štambuk and Konjevoda, 2017) [5].
Data source location	DOI link: https://doi.org/10.3390/info8010029 DOI link: https://doi.org/10.1073/pnas.1507565112
Data accessibility	With the article
Related research article	Author's names: Nikola Štambuk and Paško Konjevoda Title: Determining amino acid scores of the genetic code table: complementarity, structure, function and evolution. Journal: Biosystems 187 (2020) 104026. DOI: https://doi.org/10.1016/j.biosystems.2019.104026

## Value of the data

• The data are useful since it is shown that the nucleobase coding of amino acid hydrophobicity, specified by the 2nd codon base, is temperature independent at 25 °C and 100 °C. The hydrophobicity parameter—logarithmic equilibrium constant (Log_10_ K_w>c_) discriminates between artificial α- and β-protein datasets at 25 °C and 100 °C.
• Researchers in the areas of biochemistry and biological engineering can benefit from these data.
• The data can be used for temperature independent design of interacting peptide structures based on polar-nonpolar and neutral-neutral clustering of amino acid pairs specified by their sense and antisense (complementary) nucleobases.
• The data presented can be used for theoretical analyses of proteins, experiments with sense and antisense peptide binding, and research of biological systems at different temperature conditions.

## 1 Data description

The data presented here describe the analysis of temperature dependence of amino acid hydrophobicity parameter—Log_10_ K_w>c_ [1], with respect to the second codon base, related complementary anticodon, and artificial α- and β-protein datasets. Log_10_ K_w>c_ are logarithmic equilibrium constants for the transfer of amino acid side-chains from neutral solution to cyclohexane at 25 °C and 100 °C [1,2].

**Fig. 1:**
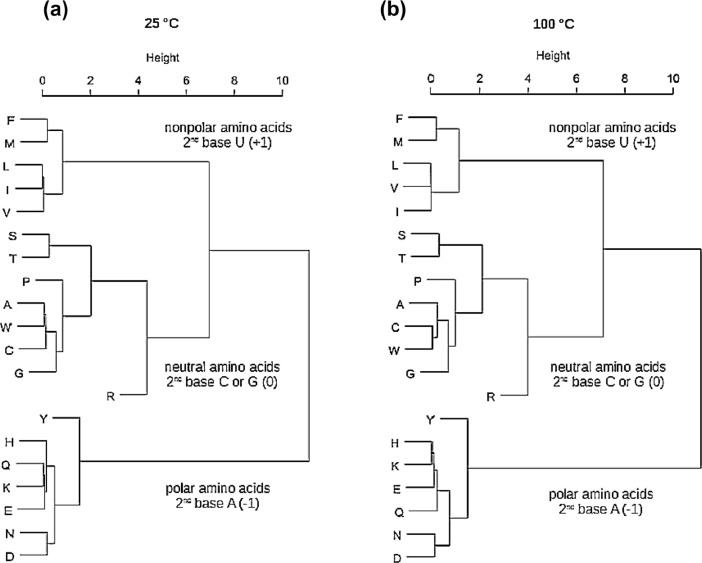
The clustering of amino acid logarithmic equilibrium constants (Log_10_ K_w>c_) for the transfer of amino acid side-chains from neutral solution to cyclohexane at 25 °C (a) and 100 °C (b) follows the amino acid partition into complementary polar-nonpolar and neutral-neutral groups associated with the second base column of the genetic code table.

Fig. 1 shows that the clustering of amino acids into polar, nonpolar and neutral groups, i.e. polar-nonpolar and neutreal-neutral clusters, is specified by the second codon base and hydrophobicity parameter—Log_10_ K_w>c_, in a temperature independent manner.

**Fig. 2:**
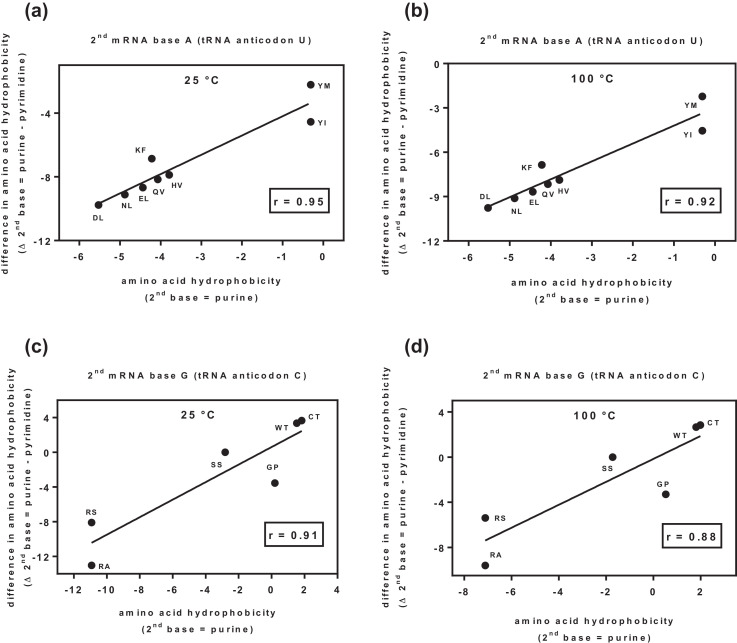
(a) Correlation of complementary pairs of *polar-nonpolar residues* in a 3′ → 5′ translation direction with respect to the logarithmic equilibrium constants (Log_10_ K_w>c_) for the transfer of amino acid side-chains from neutral solution to cyclohexane at 25 °C; (b) Correlation of complementary pairs of *polar-nonpolar residues* in a 3′ → 5′ translation direction with respect to the logarithmic equilibrium constants (Log_10_ K_w>c_) for transfer of amino acid side-chains from neutral solution to cyclohexane at 100 °C; (c) Correlation of complementary pairs of *neutral-neutral residues* in a 3′ → 5′ translation direction with respect to the logarithmic equilibrium constants (Log_10_ K_w>c_) for the transfer of amino acid side-chains from neutral solution to cyclohexane at 25 °C; (d) Correlation of complementary pairs of *neutral-neutral residues* in a 3′ → 5′ translation direction with respect to the logarithmic equilibrium constants (Log_10_ K_w>c_) for the transfer of amino acid side-chains from neutral solution to cyclohexane at 100 °C. x = free energy ligand_aa_, y = |ligand_aa_ − receptor_aa_| free energy absolute difference; r value represents Pearson correlation.

Table 1 and Fig. 2 show that the Log_10_ K_w>c_ values of the complementary, i.e. sense and antisense, amino acid pairs depend strongly on the central (2nd) purine base of the mRNA codon and the complementary pyrimidine of the tRNA anticodon. All calculated correlations are strong (*r* ≥ 0.85). With respect to the Log_10_ K_w>c_ measurements observed, temperatures of 25 °C and 100 °C do not affect the result (Table 1, Fig. 2).

**Table 1:** Correlation of complementary amino acid (aa) pairs in both 3′ → 5′ and 5′ → 3′ translation directions with respect to the logarithmic equilibrium constants (Log_10_ K_w>c_) for the transfer of amino acid side-chains from neutral solution to cyclohexane at 25 °C and 100 °C [1]. x = ligand_aa_ = amino acid Log_10_ K_w>c_ (2nd base purine or pyrimidine), y = |ligand_aa_ − receptor_aa_| = absolute difference in amino acid Log_10_ K_w>c_ at 25 °C and 100 °C (2nd base purine or pyrimidine).

Complementary aa pairs (translation direction)	polar-nonpolar (3′→5′)	neutral-neutral (3′→5′)	polar-nonpolar (5′→3′)	neutral-neutral (5′→3′)
*2nd purine base*				
Log_10_ K_w>c_ (25 °C)	0.95*	0.91*	0.90*	0.89*
Log_10_ K_w>c_ (100 °C)	0.92*	0.88*	0.85*	0.85*
*2nd pyrimidine base*				
Log_10_ K_w>c_ (25 °C)	0.72	0.44	0.38	0.37
Log_10_ K_w>c_ (100 °C)	0.77	0.51	0.56	0.45

⁎ *p* < 0.05 (Pearson r).

**Fig. 3:**
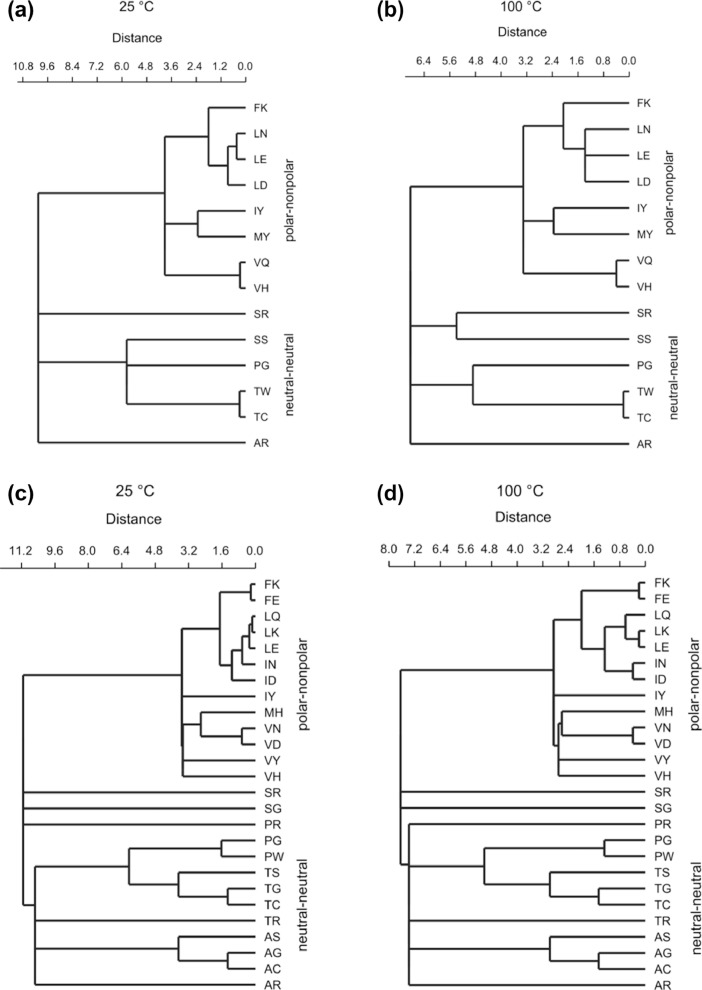
The logarithmic equilibrium constants (Log_10_ K_w>c_) for the transfer of amino acid side-chains from neutral solution to cyclohexane at 25 °C and 100 °C form two distinct, temperature independent, clusters composed of complementary pairs of polar-nonpolar and neutral-neutral residues. (a) Complementary pairs of the Log_10_ K_w>c_ values—translated in 3′ → 5′ direction at 25 °C, *r* = 0.73; (b) Complementary pairs of the Log_10_ K_w>c_ values—translated in 3′ → 5′ direction at 100 °C, *r* = 0.71; (c) Complementary pairs of the Log_10_ K_w>c_ values—translated in 5′ → 3′ direction at 25 °C, *r* = 0.62; (d) Complementary pairs of the Log_10_ K_w>c_ values—translated in a 5′ → 3′ direction at 100 °C, *r* = 0.60.

In Fig. 3 the logarithmic equilibrium constants (Log_10_ K_w>c_) specify polar-nonpolar and neutral-neutral clusters for all possible complementary codon pairs irrespective of temperature value and direction of sequence translation.

**Fig. 4:**
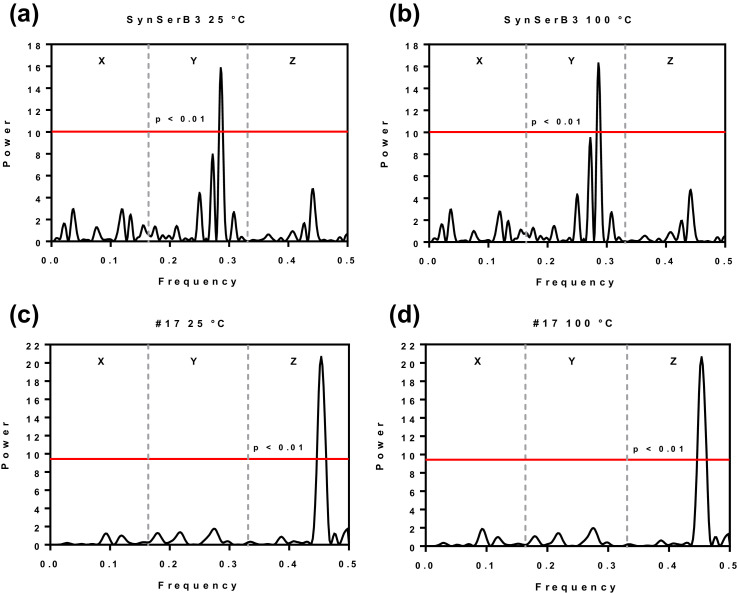
Least-squares spectral analysis of artificial Hecht_α protein SynSerB3 (a, b) [3,5] and Hecht_β protein #17 (c, d) [4,5]. The spectral analysis is based on the logarithmic equilibrium constants (Log_10_ K_w>c_) for the transfer of amino acid side-chains from neutral solution to cyclohexane at 25 °C (a, c) and 100 °C (b, d) [1,^2^,5].

Folding type predictions for 32 α- and β-artificial proteins designed by Michael Hecht and coworkers [3–5] show that the spectral analyses based on the logarithmic equilibrium constants (Log_10_ K_w>c_) accurately predict the α- and β-artificial protein class at 25 °C and 100 °C (Fig. 4). At 25 °C and 100 °C all α-proteins are characterized by a dominant peak, x = 0.29, within Y periodogram region, while all β-proteins are characterized by a dominant peak, x = 0.45, within Z periodogram region (Fig. 4). There is no difference between the 25 °C and 100 °C periodograms of the artificial proteins obtained using the Log_10_ K_w>c_ (Fig. 4). These data are confirmed using spectral analyses [6] based on Eisenberg's hydrophobic moment.

Finally, Phase 1 (primary) and Phase 2 (secondary) amino acids are clearly separated based on temperature independence of Log_10_ K_w>c_ values, and Mean Buried Area parameter [7]. The machine learning algorithm PART extracts two simple rules that correctly classify 19 out of 20 amino acids into evolutional Phase 1 (L, I, V, S, P, T, A, D, E, G) and Phase 2 (F, M, Y, H, Q, N, K, C, W, R) amino acid classes—at 25 °C and 100 °C:

PART decision list (25 °C)

———————————

IF Mean Buried Area > 97.8 Å^2^ AND Log_10_ K_w>c_ at 25 °C <= 2.64 AND Mean Buried Area > 113.9 Å^2^
THEN aa Prebiotic Phase 2 (9: F,M,Y,H,Q,K,C,W,R)
ELSE aa Prebiotic Phase 1 (11/1: L,I,V,S,P,T,A,N,D,E,G)

PART decision list (100 °C)

———————————-

IF Mean Buried Area > 97.8 Å^2^ AND Log_10_ K_w>c_ at 100 °C <= 2.60 AND Mean Buried Area > 113.9 Å^2^
THEN aa Prebiotic Phase 2 (9: F,M,Y,H,Q,K,C,W,R)
ELSE aa Prebiotic Phase 1 (11/1: L,I,V,S,P,T,A,N,D,E,G)

## 2 Experimental design, materials, and methods

### 2.1 Clustering of amino acids into polar, nonpolar and neutral groups (HAC)

The agglomerative hierarchical clustering (HAC) method in Fig. 1, consisting of the two input variables, was carried out using S-Plus 2000 software (Manhattan metric, Ward method and standardized variables). The first variable was the amino acid logarithmic equilibrium constant (Log_10_ K_w>c_, Table 1), and the second variable was the amino acid codon scoring according to Davis [8]:

• nonpolar or hydrophobic amino acid (2nd U codon score) = +1,
• polar or hydrophilic amino acid (2nd A codon score) = -1,
• neutral or intermediate amino acid (2nd C or G codon score) = 0.

High agglomerative coefficients of 0.96 were observed for 25 °C and 100 °C measurements.

### 2.2 Clustering of amino acids into polar, nonpolar and neutral groups (Fuzzy partitioning)

Two-variable fuzzy partitioning was produced using S-Plus 2000 software (*k* = 3 clusters, Manhattan metric and standardized variables). The first variable involved the amino acid logarithmic equilibrium constants (Log_10_ K_w>c_), and the second variable was amino acid codon scoring according to Davis [8] (see Section 2.1*.*). The values of average silhouette widths for 25 °C (0.67) and 100 °C (0.65) confirm the validity of the model [9], and the results of agglomerative hierarchical clustering of amino acids into polar, nonpolar and neutral groups (HAC, Fig. 1). Silhouette value measures how similar an amino acid is to its own cluster (cohesion) compared to other clusters (separation). The data show temperature independent partitioning of amino acid groups into three complementary clusters of the Standard Genetic Code Table [2]: nonpolar amino acid cluster is specified by the 2nd codon letter U (F, L, I, M, V), neutral amino acid cluster is specified by the 2nd codon letters C and G (S, P, T, A, C, W, R, G) and polar amino acid cluster is specified by the 2nd codon letter A (Y, H, Q, N, K, D, E).

### 2.3 Correlation of complementary amino acid pairs in both translation directions considering logarithmic equilibrium constants (Log_10_K_w__>c_) and temperature

Correlations of complementary pairs of polar-nonpolar residues and neutral-neutral residues in a 3′ → 5′ and 5′ → 3′ translation directions, with respect to the logarithmic equilibrium constants (Log_10_ K_w>c_) for transfer of amino acid side-chains from neutral solution to cyclohexane at 25 °C and at 100 °C. The correlations are presented in Table 1 and Fig. 2. Pearson correlation (r) was calculated for x and y variables using PAST software 3.16 (https://folk.uio.no/ohammer/past/): x = free energy ligand_aa_, and y = |ligand_aa_ − receptor_aa_| free energy absolute difference (aa = amino acid).

### 2.4 Hierarchical clustering of complementary amino acid pairs translated in both directions using Log_10_K_w__>c_ values

Constrained hierarchical clustering in Fig. 3 was produced with PAST software 3.16 using an unweighted pair-group average algorithm (UPGMA) and Euclidean similarity index. Fig. 3a and b represent the clustering of complementary amino acid pairs translated in 3′ → 5′ direction using Log_10_ K_w>c_ values at 25 °C and 100 °C, respectively. Fig. 3c and d represent the clustering of complementary amino acid pairs translated in 5′ → 3′ direction using Log_10_ K_w>c_ values at 25 °C and 100 °C, respectively.

### 2.5 Spectral analysis of artificial Hecht α and β-protein folds based on Log_10_K_w__>c_

Primary amino acid sequences of 15 artificial Hecht α- and 17 β-protein folds were converted into a numerical series by assigning the Log_10_ K_w>c_ and Eisenberg's hydrophobic moment value to each amino acid [1,^5^,6]. The Log_10_ K_w>c_ datasets of 32 α- and β-artificial protein sequences at 25 °C and 100 °C are given in Supplementary Table S1 and Supplementary Table S2. α-protein folds are numerical series 1–15 and β-protein folds are numerical series 16–32. Corresponding amino acid sequences are listed in Table S1 and Table S2 of Štambuk and Konjevoda (2017) [5]. Least-squares spectral analysis of artificial Hecht proteins, presented in Fig. 4, were carried out with PAST software 3.16. Vertical dotted lines in Fig. 4 divide the frequency axes of periodograms into three equally spaced zones: X (0–0.166), Y (0.167–0.333) and Z (0.334–0.500) [5].

### 2.6 Prediction of Phase 1 (primary) and Phase 2 (secondary) amino acids

The prediction of Phase 1 (primary) and Phase 2 (secondary) amino acids based on temperature independence of Log_10_ K_w>c_ values, and Mean Buried Area parameter [2,7] was performed using PART algorithm implemented as a part of Weka data mining software (version 3.6.13, https://www.cs.waikato.ac.nz/ml/weka/) [2].

## Appendix Supplementary materials

Image, application 1
